# Association of vitamin D_2_ and D_3_ with type 2 diabetes complications

**DOI:** 10.1186/s12902-020-00549-w

**Published:** 2020-05-15

**Authors:** Lina H. M. Ahmed, Alexandra E. Butler, Soha R. Dargham, Aishah Latif, Amal Robay, Omar M. Chidiac, Amin Jayyousi, Jassim Al Suwaidi, Ronald G. Crystal, Stephen L. Atkin, Charbel Abi Khalil

**Affiliations:** 1 Weill Cornell Medicine-Qatar, PO Box 24144, Doha, Qatar; 2 Diabetes Research Center (DRC), Qatar Biomedical Research Institute (QBRI), Hamad Bin Khalifa University (HBKU), Qatar Foundation (QF), PO Box 34110, Doha, Qatar; 3 Antidoping Laboratory Qatar, PO Box 27775, Doha, Qatar; 4 Hamad Medical Corporation, PO Box 3050, Doha, Qatar; 5 Department of Genetic Medicine, Weill Cornell Medicine, New York, USA; 6 Royal College of Surgeons Ireland, Busaiteen, Bahrain

**Keywords:** Vitamin D, Vitamin D metabolites, Type 2 diabetics, Diabetic complications, Vitamin D deficiency, Vitamin D_2_, Vitamin D_3_

## Abstract

**Aims:**

Vitamin D measurement is a composite of vitamin D_2_ (25(OH)D_2_) and D_3_ (25(OH)D_3_) levels, and its deficiency is associated with the development of type 2 diabetes (T2DM) and diabetic complications; vitamin D deficiency may be treated with vitamin D_2_ supplements. This study was undertaken to determine if vitamin D_2_ and D_3_ levels differed between those with and without T2DM in this Middle Eastern population, and the relationship between diabetic microvascular complications and vitamin D_2_ and vitamin D_3_ levels in subjects with T2DM_._

**Methods:**

Four hundred ninety-six Qatari subjects, 274 with and 222 without T2DM participated in the study. Plasma levels of total vitamin D_2_ and D_3_ were measured by LC-MS/MS analysis.

**Results:**

All subjects were taking vitamin D_2_ and none were taking D_3_ supplements. Vitamin D_2_ levels were higher in diabetics, particularly in females, and higher levels were associated with hypertension and dyslipidemia in the diabetic subjects (*p* < 0.001), but were not related to diabetic retinopathy or nephropathy. Vitamin D_3_ levels measured in the same subjects were lower in diabetics, particularly in females (*p* < 0.001), were unrelated to dyslipidemia or hypertension, but were associated with retinopathy (*p* < 0.014). Neither vitamin D_2_ nor vitamin D_3_ were associated with neuropathy. For those subjects with hypertension, dyslipidemia, retinopathy or neuropathy, comparison of highest with lowest tertiles for vitamin D_2_ and vitamin D_3_ showed no difference.

**Conclusions:**

In this Qatari cohort, vitamin D_2_ was associated with hypertension and dyslipidemia, whilst vitamin D_3_ levels were associated with diabetic retinopathy. Vitamin D_2_ levels were higher, whilst vitamin D_3_ were lower in diabetics and females, likely due to ingestion of vitamin D_2_ supplements.

## Introduction

Vitamin D measurement is a composite of both vitamin D_2_ (25(OH)D_2_) and D_3_ (25(OH)D_3_) levels. Vitamin D deficiency has been suggested to increase the risk of type 2 diabetes (T2DM) with an inverse relationship between vitamin D levels and the onset of diabetics seen in a large prospective study [1], though in a study in prediabetes patients 4000 IU daily of vitamin D_3_ supplementation did not significantly lower the risk of diabetes [2]. Deficiency of vitamin D is associated with both insulin resistance and beta cell dysfunction [3]. Whilst vitamin D deficiency in T2DM has also been correlated with microvascular complications [4], it is unclear if the deficiency is causal [4].

Vitamin D_3_ (cholecalciferol) is endogenously produced in the skin through the effect of UV-B on 7-dehydrocholesterol, whilst vitamin D_2_ is derived from the diet as ergosterol, primarily from mushrooms and fungi, and then converted to ergocalciferol by ultraviolet light; both are hydroxylated at position 25 to 25hydroxy vitamin D_2_ (25(OH)D_2_) or 25hydroxy vitamin D_3_ (25(OH)D_3_). 25(OH)D is transported to the kidney and converted to the active 1,25-dihydroxyvitamin D by 1 alpha hydroxylase; however, 1,25-dihydroxyvitamin D is produced in extrarenal tissues and may act locally. In most countries, vitamin D_2_ is available as a pharmaceutical and a supplement to counter vitamin D deficiency.

While vitamin D deficiency is a global issue, it is a particular problem in the Middle East [5]. Additionally, over 20% of the Qatari population has T2DM, a prevalence two-fold higher than the world average.

This study was undertaken to determine the relationship of vitamin D_2_ (25(OH)D_2_) and vitamin D_3_ (25(OH)D_3_) levels between subjects with and without T2DM in this Middle Eastern population, and to determine the microvascular complications in this vitamin D deficient Qatari population with and without T2DM. We further hypothesized that, in the T2DM cohort, vitamin D_2_ would mirror vitamin D_3_ levels and both would be associated with the same diabetic complications.

## Methods

### Study population

Four hundred ninety-six Qatari subjects, 274 with and 222 without T2DM, participated in the study. Diabetic subjects were recruited from Hamad General Hospital diabetics clinic, and nondiabetic subjects were comprised of relatives accompanying the T2DM subjects between July 2013 and 2015 (Table 1).

**Table 1:** Demographic data (mean (SD)), vitamin D_2_ and vitamin D_3_ levels in the Control (*n* = 222) and Type 2 Diabetic (*n* = 274) cohorts

	Control	Diabetes	*P*-value
	Median (Range)	Median (Range)	
**Age (years)**	46.1 (10.8)	55.2 (9.9)	< 0.001
**BMI (kg/m**^**2**^**)**	30.1 (34.8)	32.4 (44.0)	< 0.001
**HbA1c (%)**	5.6 (4.6)	7.9 (11.2)	< 0.001
**Glucose (mmol/L)**	5.2 (14.6)	8.6 (26.7)	< 0.001
**Duration of diabetes (years)**		10.6 (6.5)	
**25(OH)D**_**3**_**(ng/dl)**	8.824 (55.599)	6.450 (55.475)	< 0.001
**25(OH)D**_**2**_**(ng/dl)**	7.867 (59.113)	19.428 (57.014)	< 0.001

Inclusion criteria were male or female Qataris, aged 30 years or older. The diagnosis of T2DM was made according to WHO guidelines. Inclusion in the nondiabetic control group required a normal glucose tolerance test. Exclusion criteria were a diagnosis of type 1 diabetes, gestational diabetes or diabetes secondary to steroid treatment. All diabetic subjects had retinal photography, a clinical foot examination and blood pressure measurement.

All subjects had been prescribed vitamin D_2_ supplements 50,000 units weekly that they had been taking for more than 6 months, and none were taking vitamin D_3_ supplements: normal controls were taking no medication.

The study was approved by Weill Cornell IRB (IRB# 13–00063) and all participants provided written informed consent. The conduct of the trial was in accordance with ICH GCP and the Declaration of Helsinki.

### Study design

Fasting blood samples were collected, and weight and blood pressure measured, at the baseline visit. Samples were separated by centrifugation at 2000 *g* for 15 min at 4 °C, and stored at − 80 °C within 1 h of collection. Blood pressure was measured using an automated device (NPB-3900; Nellcor Puritan Bennett, Pleasanton, CA) at rest. Dyslipidemia was defined as a total cholesterol > 190 mg/dl (> 4.9 mmol/l) and/or fasting triglycerides > 150 mg/dl (> 1.7 mmol/l) untreated, or if subjects were under treatment. Diabetic retinopathy was diagnosed by fundoscopy. Diabetic neuropathy was diagnosed based by vibration perception threshold (Neurothesiometer NU-1, Horwell- UK) of the great toe being > 25 V.

### Serum vitamin D_2_ and vitamin D_3_ measurement

Vitamin D measurement was undertaken as follows: Serum 25(OH)D levels were quantified using isotope-dilution liquid chromatography tandem mass spectrometry (LC-MS/MS). Twenty-five μL of internal standards d6-25OHD_3_ (50 ng/mL) and d6-25OHD_2_ (20 ng/mL) were added into each microcentrifuge tube containing 250 μL of calibration standards, Quality Control or serum samples, and kept for 30 min to reach binding equilibrium. The samples were diluted with 250 μL of isopropanol and water, 50:50 v/v, and left to stand for at least 15 min to displace binding protein.

Three hundred μL of pre-treated samples were loaded onto ISOLUTE® supported liquid extraction (SLE+) columns (Biotage), followed by elution with 1.8 mL of n-heptane (2 × 900 μL) into a collection tube already containing 200 μL of 0.25 mg/mL PTAD solution in ethyl acetate and heptane (8:92 v/v). The eluate was evaporated to dryness using turbovap under nitrogen gas heated at 38 °C. Once dried, 50 μL of reconstituted solution consisting of methanol and deionized water, 70:30 v/v, and 0.006% methylalamine were added into all tubes. The derivatised extracts were transferred into LC insert vials and 10 μL from each was injected into the LC-MS/MS system.

### Study outcomes

#### Statistical analyses

The sample size of this study was based on another that found 51% of diabetics without microvascular complications and 80% with retinopathy had vitamin D deficiency [4]. Using the 49% without retinopathy as the comparison group, a sample size of 274 diabetic patients was selected and provides 80% power to detect a 68% prevalence of vitamin D deficiency in the retinopathy group. When examining mean vitamin D differences here, the 274 patients, assuming 40% (*N* = 110) have retinopathy, yields a harmonic mean of the sample size of about 132. This sample size provides 80% power for a difference in vitamin D means of 0.35 deviations using a t-test, considered a moderate-sized effect.

Data trends were visually and statistically evaluated for normality. Non-parametric tests (Mann Whitney U) were applied on data that violated the assumptions of normality when tested using the Kolmogorov-Smirnov Test. Bonferroni correction was applied to account for multiple testing. Statistical analysis was performed using SPSS for Windows, version 24.0. All values are given as mean ± SD or as mean with 95% confidence interval (CI) unless otherwise specified. Correlations between vitamin D_2_ and D_3_ were undertaken with Spearman’s correlation.

## Results

### Baseline characteristics

The baseline characteristics for the T2DM and control cohorts are shown in Table 1. The relationship of glycemic control for both HbA1c and blood glucose at the time of the visit for T2DM are shown in Table 2, showing that only HbA1c was significantly different in retinopathy (*p* < 0.001) whilst blood glucose was different in hypertension (*p* < 0.02).

**Table 2:** The relationship of glycemic control for HbA1c and blood glucose in T2DM subjects

	HbA1c		Glucose	
	Median (IQR)	*P*-value	Median (IQR)	*P*-value
**Hypertension-No**	7.7 (6.7–9.4)	0.730	8.9 (6.6–12.8)	0.023
**Hypertension-Yes**	8.0 (6.7–9.3)		7.8 (6.1–10.6)	
**Dyslipidemia-No**	7.7 (6.6–9.2)	0.360	8.5 (6.3–13.1)	0.419
**Dyslipidemia-Yes**	8.0 (6.8–9.4)		8.2 (6.2–10.7)	
**Retinopathy-No**	7.4 (6.6–9.1)	0.001	8.1 (6.3–10.7)	0.599
**Retinopathy-Yes**	8.6 (7.3–9.8)		8.6 (6.2–10.9)	
**Neuropathy-No**	7.8 (6.7–9.2)	0.119	8.2 (6.2–10.8)	0.325
**Neuropathy-Yes**	8.2 (7.2–9.7)		8.5 (6.7–11.3)	
**PAD-No**	7.9 (6.7–9.3)	0.970	8.3 (6.2–10.8)	0.757
**PAD-Yes**	7.3 (6.7–9.7)		7.8 (7.2–14.9)	
**CAD-No**	7.8 (6.7–9.3)	0.287	8.4 (6.3–10.9)	0.645
**CAD-Yes**	8.1 (6.9–9.9)		8.1 (6.2–9.5)	
**Stroke-No**	7.8 (6.7–9.3)	0.297	8.3 (6.1–10.9)	0.576
**Stroke-Yes**	8.8 (6.9–9.8)		8.5 (7.1–10.9)	

*PAD* Peripheral artery disease, *CAD* coronary artery disease

#### Vitamin D measurements

Vitamin D_2_ levels were higher in diabetes, particularly in females, and higher levels were associated with hypertension and dyslipidemia in the diabetic subjects (*p* < 0.001), but were not related to diabetic retinopathy or nephropathy. Vitamin D_3_ levels measured in the same subjects were lower in diabetes, particularly in females (*p* < 0.001), were unrelated to dyslipidemia or hypertension, but were associated with retinopathy (*p* < 0.014). Neither vitamin D_2_ nor vitamin D_3_ were associated with neuropathy (Table 3). The Endocrine Society defines vitamin D deficiency, insufficiency and repletion as ≤20 ng/mL, 20-30 ng/mL and ≥ 30 ng/mL, respectively. Comparison of those T2DM who were vitamin D deplete compared those who were vitamin D replete for both vitamin D_2_ and vitamin D_3_ levels showed no difference for hypertension, dyslipidemia, retinopathy or neuropathy.

**Table 3:** The relationship of Vitamin D_2_ and D_3_ to diabetes complications in the cohort of subjects with Type 2 Diabetes (*n* = 274). The actual number of diabetic patients per measure are detailed and may not add up to 274 due to missing values when the vitamin D levels were at the lower limit of detection then as they could not be accurately determined they were excluded from the analysis

	25(OH)D_**2**_ (ng/dl) median (range)	*P*-value	25(OH)D_3_ (ng/dl) median (range)	*P*-value
**Diabetes**				
No *n* = 222	7.85 (20.5)	< 0.001	8.95 (9.31)	< 0.001
Yes *n* = 274	20.2 (21.1)		6.21 (10.53)	
**Gender**				
Male *n* = 110	14.07 (21.69)	< 0.001	8.26 (11.01)	< 0.001
Female *n* = 164	23.06 (21.31)		4.52 (7.44)	
**Hypertension**				
No *n* = 96	13.82 (22.14)	< 0.001	6.39 (10.39)	0.612
Yes *n* = 163	21.63 (20.57)		6.06 (10.61)	
**Dyslipidemia**				
No *n* = 71	11.80 (18.58)	< 0.001	6.86 (10.63)	0.061
Yes *n* = 188	21.58 (18.95)		5.59 (9.0)	
**Diab_Retinopathy**				
No *n* = 182	19.53 (20.28)	0.445	6.25 (8.96)	0.014
Yes *n* = 77	20.65 (21.78)		7.98 (11.76)	
**Diab_Neuropathy**				
No *n* = 209	19.42 (20.99)	0.517	6.25 (10.66)	0.74
Yes *n* = 50	21.52 (19.59)		6.18 (7.79)	

In control subjects, vitamin D_2_ was higher in those with both hypertension and dyslipidemia (*p* < 0.02) as was seen for the patients with T2DM, whereas vitamin D_3_ was lower in controls with dyslipidemia (*p* < 0.001), an association not seen in T2DM subjects (Table 4).

**Table 4:** The relationship of vitamin D_2_ and D_3_ to hypertension and dyslipidemia in control subjects

	Vitamin D_2_			Vitamin D_3_	
	Median (IQR)	Mean (SD)	*P*-value	Median (IQR)	*P*-value
**Hypertension-No**	0.00 (0.00–0.00)	9.07 (11.09)	0.022	0.17 (0.00–0.59)	0.350
**Hypertension-Yes**	0.00 (0.00–0.00)	15.28 (15.65)		0.19 (0.00–0.78)	
**Dyslipidemia-No**	0.00 (0.00–0.00)	7.58 (10.04)	< 0.001	0.20 (0.00–0.79)	< 0.001
**Dyslipidemia-Yes**	0.00 (0.00–0.00)	16.37 (14.82)		0.13 (0.00–0.36)	

The relationship of vitamin D_2_ and D_3_ to diabetic complications according to gender is shown in Table 5 that shows significant differences between the genders: for hypertension, dyslipidemia and neuropathy for vitamin D_2_ and retinopathy and dyslipidemia for vitamin D_3_.

**Table 5:** The relationship of vitamin D_2_ and vitamin D_3_ to diabetic complications according to gender

		Females					
		Vit D_2_			Vit D_3_		
		Mean (SD)	Median (IQR)	P	Mean (SD)	Median (IQR)	P
**Hypertension**	**No**	19.6 (12.6)	20.1 (9.9–27.2)	0.058	7.5 (7.8)	4.6 (2.8–8.2)	0.838
	**Yes**	24.5 (14.7)	23.1 (14.7–36.5)		8.6 (9.0)	4.5 (2.7–10.2)	
**Dyslipidemia**	**No**	19.9 (13.8)	16.8 (8.9–29.7)	0.086	8.4 (8.4)	5.5 (3.6–8.3)	0.294
	**Yes**	24.0 (14.2)	23.2 (14.8–35.0)		8.1 (8.8)	4.2 (2.6–10.2)	
**Retinopathy**	**No**	22.8 (13.8)	21.4 (11.7–33.4)	0.726	7.3 (8.2)	3.9 (2.5–7.4)	0.007
	**Yes**	23.2 (15.3)	25.5 (13.5–35.5)		10.5 (9.2)	6.8 (3.5–15.8)	
**Neuropathy**	**No**	22.7 (14.4)	22.0 (11.2–33.2)	0.554	8.0 (8.7)	4.3 (2.6–9.7)	0.051
	**Yes**	24.3 (13.4)	23.1 (15.8–36.5)		9.6 (8.1)	6.6 (3.7–16.7)	
**PAD**	**No**	23.3 (13.9)	22.8 (13.0–34.1)	0.135	8.1 (8.5)	4.4 (2.7–9.8)	0.607
	**Yes**	13.3 (18.3)	2.8 (0.5–32.1)		10.8 (11.8)	5.9 (3.3–18.6)	
**CAD**	**No**	22.4 (13.7)	22.4 (11.7–32.9)	0.422	8.2 (8.5)	4.4 (2.7–10.3)	0.939
	**Yes**	25.6 (16.7)	22.4 (16.1–37.6)		8.1 (9.3)	4.6 (2.8–7.1)	
**Stroke**	**No**	22.8 (14.2)	21.6 (11.7–33.8)	0.488	8.3 (8.6)	4.5 (2.8–10.2)	0.357
	**Yes**	25.9 (14.5)	25.3 (19.4–39.7)		7.1 (10.1)	4.5 (0.9–7.1)	
		**Males**					
		**Vit D**_**2**_			**Vit D**_**3**_		
		**Mean (SD)**	**Median (IQR)**	**P**	**Mean (SD)**	**Median (IQR)**	**P**
**Hypertension**	**No**	10.5 (11.1)	5.7 (0.6–20.6)	0.04	11.4 (8.4)	9 (4.6–15.6)	0.313
	**Yes**	15.3 (12.2)	15.6 (2.8–25.1)		9.6 (6.9)	7.8 (4.2–14.7)	
**Dyslipidemia**	**No**	7.9 (8.2)	5.2 (1.4–12.3)	0.006	13 (8.8)	10.2 (7–17.6)	0.026
	**Yes**	15.3 (12.6)	16 (2.4–25.7)		9.4 (6.8)	6.9 (4.1–15.3)	
**Retinopathy**	**No**	12.8 (12.3)	10 (0.6–23.8)	0.368	10.5 (7.8)	8.2 (4.3–15.6)	0.897
	**Yes**	14.4 (11.4)	13.5 (3.6–20.6)		10 (7)	8.3 (4.4–15.4)	
**Neuropathy**	**No**	12.2 (11.9)	9.9 (0.8–21.8)	0.029	10.8 (7.4)	8.7 (4.6–16.3)	0.075
	**Yes**	17.2 (11.8)	19.9 (5.3–25)		8.7 (7.9)	5.9 (3.6–11.4)	
**PAD**	**No**	13.3 (12.2)	11.8 (1.1–23.8)	0.704	10.4 (7.7)	8 (4.4–15.6)	0.996
	**Yes**	13.3 (9.3)	16 (2.9–21.8)		9.9 (5.4)	11.2 (2.9–13.6)	
**CAD**	**No**	12.9 (12.1)	10 (1.1–22.7)	0.335	10.5 (7.6)	8.3 (4.3–15.6)	0.604
	**Yes**	15.7 (11.4)	17.2 (3.9–23.8)		9.5 (7.1)	6 (4.8–15.4)	
**Stroke**	**No**	13.2 (12)	11.8 (1.4–22.6)	0.342	10.4 (7.6)	8.2 (4.6–15.4)	0.821
	**Yes**	17 (11.9)	18.9 (5.6–22.7)		9.4 (6.7)	10.4 (2.9–12)	

*PAD* peripheral artery disease, *CAD* coronary artery disease

## Discussion

This study was specifically powered on vitamin D and retinopathy and showed that vitamin D_3_ but not vitamin D_2_ was associated with diabetes retinopathy. Further, the levels of vitamin D_2_ were higher in diabetes and in females, likely reflecting ingestion of vitamin D_2_ supplements by these subjects; all subjects had been prescribed vitamin D_2_ 50,000 units weekly. The increased prevalence of hypertension and dyslipidemia seen in diabetes would reflect on the elevated vitamin D_2_ levels seen rather than a causal association and this was also seen in the control population; this was confirmed when deficient and replete vitamin D_2_ populations were compared and no difference found between them for hypertension or dyslipidemia. There was no association with either diabetic retinopathy or neuropathy complications. When gender differences were investigated, significant differences were seen in male T2DM for hypertension, dyslipidemia and neuropathy with vitamin D_2_ and for dyslipidemia with vitamin D_3_, whilst a difference for retinopathy was seen for female T2DM patients not seen in males. This may be of particular importance, as lower vitamin D levels in women have been associated with increased severity of coronary artery disease [6], but the impact of gender differences in diabetes complication onset or severity with respect to vitamin D deficiency is unknown.

Patients were vitamin D_3_ supplement naïve and levels of vitamin D_3_ were lower in diabetes and in females and hypertension, dyslipidemia or diabetic neuropathy complications did not differ; however, higher vitamin D_3_ levels were associated with diabetic retinopathy though patients still had an overall vitamin D_3_ deficiency. This association with total vitamin D was shown in two systematic reviews and meta-analysis of over 14 studies of 10,000 patients, that showed the statistical significant association between vitamin D deficiency and diabetic retinopathy [7, 8]. However, this has not been found in other studies [9]. It is unclear whether vitamin D deficiency is causative, contributory or simply associated with the development of diabetic retinopathy; however, one study has suggested that vitamin D is neuroprotective for optic nerves with vitamin D deficiency associated with retinal nerve fiber layer thinning [10].

Retinopathy was associated with poorer glycemic control and with lower vitamin D_3_ levels, reflecting the literature showing poorer glycemic control is associated with lower vitamin D levels [11]. What was suggested here is that the vitamin D_3_ component of total vitamin D deficiency may be more important that the vitamin D_2_ for the development of retinopathy, but there are no data looking at this aspect. Whilst those subjects with diabetes and female subjects had lower levels of vitamin D_3_, when vitamin D_2_ and vitamin D_3_ were combined (total vitamin D), overall levels of total vitamin D remained higher in diabetes. Vitamin D_2_ and vitamin D_3_ have generally been considered to be equipotent and vitamin D_2_ and vitamin D_3_ supplements may contribute equally to the circulating total vitamin D pool [12]. However, there is evidence that the actions of vitamin D_2_ or its metabolites may have a differing action to vitamin D_3_ or its metabolites at both the molecular [13] and clinical levels [14], that may account for the difference seen in the diabetes related complications.

Vitamin D deficiency may contribute to the pathogenesis of type 2 diabetes, and epidemiological evidence links it with insulin resistance [15]. Supplementation with cholecalciferol may improve beta cell function, though no protective effect of vitamin D was found on diabetes risk, clinically [16]. Differing meta-analyses have reported an improvement in HbA1c in response to vitamin D supplementation in some, but not in others [17]. Vitamin D deficiency has been associated with development of microvascular complications in type 2 diabetes [18].

Our results showed that vitamin D_2_ levels were higher in diabetes, the converse to that expected, likely due to the ingestion of supplements; higher levels did not reflect upon diabetes complications reduction. Conversely, vitamin D_3_ levels were low, particularly in the Qatari type 2 diabetic females versus males, in accord with other studies [19], though this is not a universal finding [4]. The definition of vitamin D sufficiency is based on total vitamin D levels that are a composite of vitamin D_2_ and vitamin D_3,_ and it also raises the suggestion that differing diabetic complications may relate more to one form of vitamin D than to the other.

The strength of this study was the measurement of the vitamin D_2_ and vitamin D_3_ by state-of-the-art LC-MS in a homogeneous Qatari population. However, this is still a relatively small cohort and the cross-sectional design is a limitation. In addition, it is important to note that this study measured only levels of vitamin D_2_ and vitamin D_3_, rather than the active forms of 1,25(OH)_2_D. No analysis for age could be undertaken as the data was skewed. Furthermore, this was a cross sectional study and therefore we did not have serial measurements such as blood pressure. It has been shown that ingestion of such doses of vitamin D_2_ as given here results in steady state levels even if given monthly, though vitamin D repletion may not be achieved and may vary between individuals [20]; therefore, the timing of the dosing of vitamin D_2_ supplementation would likely not influence day to day vitamin D levels.

In conclusion, vitamin D_3_ was associated with diabetic retinopathy whilst vitamin D_2_ was not. Overall, vitamin D levels were higher overall in diabetes and females, likely due to the ingestion of vitamin D_2_ supplements; conversely, vitamin D_3_ levels were lower in diabetes and in females.

## Data Availability

The data that support the findings of this study are available from the corresponding author upon request.
